# Heterogenous pathogen profile associated with acute conjunctivitis in Nepal

**DOI:** 10.1093/inthealth/ihad079

**Published:** 2023-08-31

**Authors:** Meenu Chaudhary, Sanjeeta Sitaula, Kevin Ruder, Cindi Chen, Lina Zhong, Yu Heng Lu, Thomas Abraham, Danny Yu, Armin Hinterwirth, Thomas M Lietman, Thuy Doan, Gerami D Seitzman, the SCORPIO Study Group, Lalitha Prajna, N Venkatesh Prajna, Ramesh Gunasekaran, Sankalp Singh Sharma, Vishnu Teja, Meenu Chaudhary, Sanjeeta Sitaula, Ali Sié, Boubacar Coulibaly, Mamadou Bountogo, Thanapong Somkijrungroj, Vannarut Satitpitakul, Huy Tran, Linh Hoàng Mai, Thảo Hạ Xuân, Yen Tran, Cristhian A Urzua, Fabian Vega, Felipe Salgado, Loreto Cuitino, Fernando Pérez Pérez, Jaime Macías Martínez, Van Charles Lansingh, Sukhumal Thanapaisal, Wipada Laovirojjanakul, George McKie, Kenia Chavez, Travis Redd, Winston Chamberlain, Angel Cheng, Vivien Tham, Wiwan Sansanayudh, Abba Kaka Hajia Yakoura, Abdou Amza, Abdoul Salam Youssoufou Souley, Adam Nouhou Diori, Beido Nassirou, Boubacar Kadri, Boubacar Mariama, Cissé Mamadou Ibrahim, Lamyne Aboubacar Roufaye, Ramatou Boulhassane, Saley Ali, Zakou Abdou, Lee Goren, Ruti Sella, Clare Kelliher, Laura Green, Hon Shing Ong, Jod Mehta, Yu-Chi Liu, Benjamin A Pinsky, De-Kuang Hwang, Nai-Wen Fan, Hong Sheng Chiong, Javier Lacorzana, Maria Cabrera-Aguas, Stephanie Watson, Edmund Tsui, Nina M Cherian, Rachel Feit-Leichman, Reginald E Hughes, Tania Onclinx, Joseph K Privratsky, Carol Yu, Esmeralda McClean, Iliana Molina, Armin Hinterwirth, Cindi Chen, Danny Yu, David Liu, Elodie Lebas, Emily Colby, Gerami Seitzman, Kevin Ruder, Lina Zhong, Michael Deiner, Thomas Abraham, Thomas Lietman, Thuy Doan, Travis Porco, Stephen McLeod, Kuniyoshi Kanai, Meredith Whiteside, Steven Yeh, Tolulope Fashina, James Chodosh, Bridgit Tarkap, Jambi N Garap, Magdalene Mangot, Edwin Amel, Fasihah Taleo, Johnson Kasso, Kalbule Willie, Madopule Nanu, Prudence Rymill, Anthony W Solomon

**Affiliations:** B.P. Koira la Lions Center for Ophthalmic Studies, Institute of Medicine, Tribhuvan University, Kathmandu 44600, Nepal; B.P. Koira la Lions Center for Ophthalmic Studies, Institute of Medicine, Tribhuvan University, Kathmandu 44600, Nepal; F. I. Proctor Foundation, University of California San Francisco, San Francisco, California 94158, USA; F. I. Proctor Foundation, University of California San Francisco, San Francisco, California 94158, USA; F. I. Proctor Foundation, University of California San Francisco, San Francisco, California 94158, USA; F. I. Proctor Foundation, University of California San Francisco, San Francisco, California 94158, USA; F. I. Proctor Foundation, University of California San Francisco, San Francisco, California 94158, USA; F. I. Proctor Foundation, University of California San Francisco, San Francisco, California 94158, USA; F. I. Proctor Foundation, University of California San Francisco, San Francisco, California 94158, USA; F. I. Proctor Foundation, University of California San Francisco, San Francisco, California 94158, USA; Department of Ophthalmology, University of California San Francisco, San Francisco, California 94158, USA; F. I. Proctor Foundation, University of California San Francisco, San Francisco, California 94158, USA; Department of Ophthalmology, University of California San Francisco, San Francisco, California 94158, USA; F. I. Proctor Foundation, University of California San Francisco, San Francisco, California 94158, USA; Department of Ophthalmology, University of California San Francisco, San Francisco, California 94158, USA

**Keywords:** infectious conjunctivitis, RNA deep-sequencing, SCORPIO

## Abstract

**Background:**

Infectious conjunctivitis is common in Nepal.

**Materials and Methods:**

This prospective study recruited 60 patients with presumed acute infectious conjunctivitis from the B.P. Koirala Lions Center for Ophthalmic Studies in Kathmandu, Nepal. Swabs from the conjunctiva and anterior nares were processed for metagenomic RNA deep sequencing (RNA-seq).

**Results:**

Pathogens were identified in 55% of cases. RNA viruses were the most common pathogen class identified. Severe acute respiratory syndrome coronavirus 2 was the most common RNA virus identified.

**Conclusions:**

Acute infectious conjunctivitis varies by location. Contrary to expectations, RNA viruses predominated. Repeat surveillance may be useful and RNA-seq allows for detection of the unexpected pathogen including RNA viruses.

## Introduction

Conjunctivitis is a common health problem in Nepal. In 2003, Nepal experienced a nationwide outbreak of acute hemorrhagic conjunctivitis, affecting nearly one-half of the entire population. Both human adenovirus and coxsackievirus viruses were identified,^1^ suggesting that epidemics can be caused by more than one pathogen and that DNA and RNA viruses can co-mingle. A 2011 prospective Kathmandu-based study identified *Streptococcus pneumonia* as the most common isolated organism in young patients presenting with acute conjunctivitis.^2^ A 2013 prospective study in Pokhara identified *Staphylococcus aureus* as the most common. Viruses were not queried in either the 2011 or 2013 studies. SCORPIO—Seasonal Conjunctivitis Outbreak Reporting for Prevention and Improved Outcomes—is a University of California San Francisco (UCSF)-based international study aiming to determine worldwide causes of infectious conjunctivitis. B.P. Koirala Lions Center for Ophthalmic Studies (BPKLCOS) in Kathmandu, Nepal, is one participating site.

## Materials and Methods

This research was approved by the Institutional Review Boards of both BPKLCOS in Kathmandu, Nepal, and the UCSF, and adhered to the tenets of the Declaration of Helsinki. Samples were obtained from August 2021 through September 2022 at the BPKLCOS eye clinic. Inclusion criteria required signs and symptoms suggestive of acute infectious conjunctivitis for a duration of <14 d in any age group. Sterile polyester applicators (Puritan) were used to swab the lower fornix of each eye and each anterior nasal passage. Swabs were placed in a DNA/RNA-Shield (Zymo Research) and stored in a –20°C freezer prior to shipping to UCSF for processing. Sample processing, library preparation, sequencing and bioinformatic algorithm for pathogen identification have been previously described.^3^ The prespecified criteria for pathogen identification were: (1) organism known to be a human pathogen and representing the most abundant matched reads after water background subtraction; or for viral pathogens (2) ≥2 unique reads covering separate regions in DNA virus genomes; or (3) ≥1 unique reads matching RNA virus genomes.

## Results

**Figure 1.**
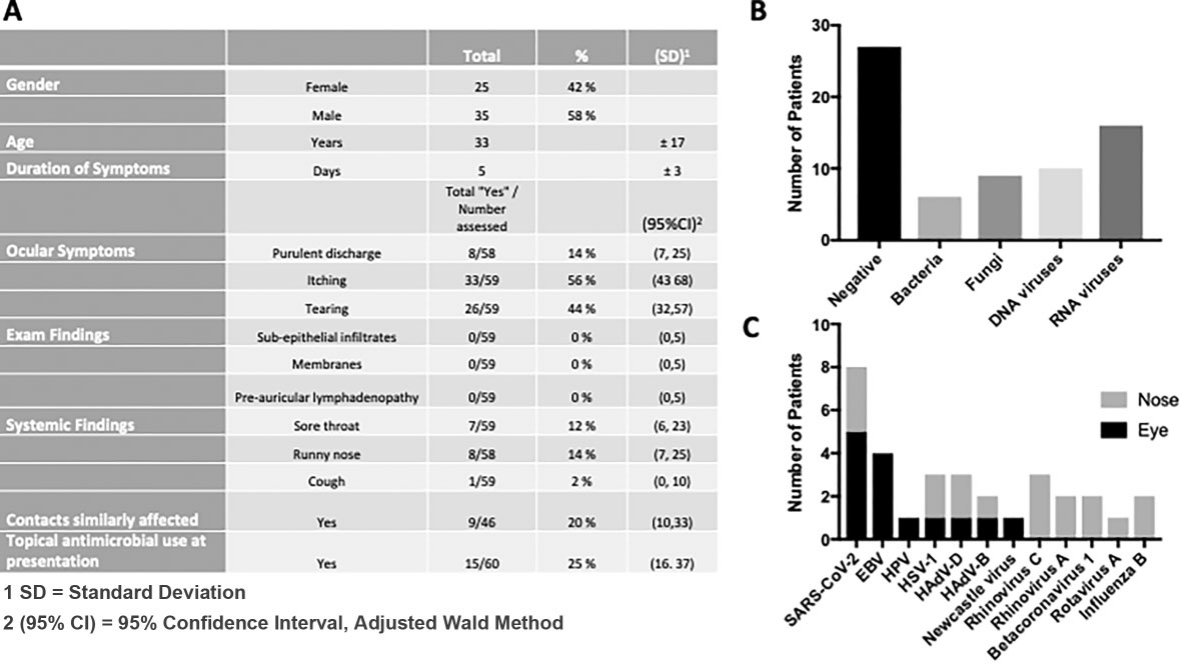
(A) Demographics and clinical signs and symptoms of study participants. (B) Pathogen class identified by RNA-seq. (C) Specific pathogens identified in conjunctiva and nasal swabs.

Sixty patients were enrolled. Forty-two percent were female. Demographics, clinical signs and symptoms are documented in Figure 1A. The mean age was 33 (SD±17) y. Bilateral eye involvement occurred in 30% (95% CI 20 to 43%). Itching was the most reported eye symptom present in 56% (95% CI 43 to 68%) followed by tearing in 44% (95% CI 32 to 57%), then purulent discharge in 14% (95% CI 7 to 25%). On clinical examination, subepithelial infiltrates were present in 19% (95% CI 11 to 31%), membranes or pseudo-membranes in 3% (95% CI 0 to 12%) and preauricular lymphadenopathy in 2% (95% CI 0 to 10%). Comorbid systemic symptoms were not common with rhinorrhoea in 14% (95% CI 7 to 25%), sore throat in 12% (95% CI 6 to 23%) and cough in 2% (95% CI 0 to 10%). Twenty percent (95% CI 10 to 33%) reported similarly affected close contacts. Twenty-eight percent (95% CI 18 to 41%) presented for care on topical antibiotics. Metagenomic sequencing identified candidate pathogens in 55% (95% CI 42 to 67%). Polymicrobial infections were suspected in 13% (95% CI 7 to 24%). Of the pathogens identified (Figures 1B and 1C), RNA viruses were the most common class of pathogens identified in 16 patients (including conjunctival and nasal swabs). Genomic reads corresponding to severe acute respiratory syndrome coronavirus 2 (SARS-CoV-2) were the most frequently isolated RNA virus, identified in five of the ocular swabs and three of the nasal swabs. Notably, two cases of common circulating betacoronavirus 1 and one case of Newcastle virus,^4^ were identified. Bacterial pathogens were associated with nine participants, with *Streptococcus pneumoniae* representing the most frequent isolate and *Haemophilus* spp. identified as the other bacterial pathogen. DNA viruses were identified in 17% of the patients. Interestingly, adenoviruses were only identified in three cases representing only 5% (95% CI 1 to 14%) of the overall case series. One fungal pathogen, *Vittaforma corneae*, was identified in six of the 60 samples, representing 10% (95% CI 4 to 20%) overall.

## Discussion

In this case series, pathogens associated with acute conjunctivitis in Katmandu Nepal are diverse. Interestingly, SARS-CoV-2 was the most frequent pathogen associated with acute infectious conjunctivitis. Even although three of the five patients in whom SARS-CoV-2 was identified from the conjunctival swabs also had this virus present in their nasal swabs, none of these participants reported any systemic symptoms. This study is limited by a lack of normal controls and no follow-up data. Like many sites in the SCORPIO study, roughly one-third of the patients presented using topical antimicrobial drops, which limit bacterial and some fungal pathogen identification. This study highlights the importance of location in disease surveillance, as different pathogens appear to predominate in different areas of the world. Repeat surveillance, especially with seasonal variation, is important. Lastly, the use of RNA-deep sequencing emphasises the importance of RNA virus detection in disease surveillance.

## Data Availability

The data underlying this article will be shared on reasonable request to the corresponding author.

## Seasonal Conjunctivitis Outbreak Reporting for Prevention and Improved Outcomes (SCORPIO) Study Group

*Aravind Eye Hospital, Madurai, India—*Lalitha Prajna, N. Venkatesh Prajna, Ramesh Gunasekaran, Sankalp Singh Sharma, Vishnu Teja; *B.P Koirala Lions Center for Ophthalmic Studies, Kathmandu, Nepal—*Meenu Chaudhary, Sanjeeta Sitaula; *Centre de Recherche en Sante de Nouna, Nouna, Burkina Faso—*Ali Sié, Boubacar Coulibaly, Mamadou Bountogo; *Chulalongkorn University, Bangkok, Thailand—*Thanapong Somkijrungroj, Vannarut Satitpitakul; *Hai Yen Vision Institute, Ho Chi Minh City, Vietnam—*Huy Tran, Linh Hoàng Mai, Thảo Hạ Xuân, Yen Tran; *Hospital Clinico Universidad de Chile, Santiago, Chile—*Cristhian A. Urzua, Fabian Vega, Felipe Salgado, Loreto Cuitino; *Instituto Mexicano de Oftalmología, Santiago de Querétaro, Mexico—*Fernando Pérez Pérez, Jaime Macías Martínez, Van Charles Lansingh; *Khon Kaen University, Khon Kaen, Thailand—*Sukhumal Thanapaisal, Wipada Laovirojjanakul; *National Eye Institute*—George McKie (Program Officer); *Oregon Health and Science University, Portland, Oregon, USA—*Kenia Chavez, Travis Redd, Winston Chamberlain; *Pacific Vision Institute of Hawaii, Honolulu, Hawaii, USA—*Angel Cheng, Vivien Tham; *Phramongkutklao Hospital, Bangkok, Thailand—*Wiwan Sansanayudh; *Programme National de Santé Oculaire, Niamey, Niger—*Abba Kaka Hajia Yakoura, Abdou Amza, Abdoul Salam Youssoufou Souley, Adam Nouhou Diori, Beido Nassirou, Boubacar Kadri, Boubacar Mariama, Cissé Mamadou Ibrahim, Lamyne Aboubacar Roufaye, Ramatou Boulhassane, Saley Ali, Zakou Abdou; *Rabin Medical Center, Petah Tikva, Israel—*Lee Goren, Ruti Sella; *Sinai Hospital, Baltimore, Maryland, USA*—Clare Kelliher, Laura Green; *Singapore Eye Research Institute, Singapore—*Hon Shing Ong, Jod Mehta, Yu-Chi Liu; *Stanford University School of Medicine*—Benjamin A. Pinsky; *Taipei Veterans General Hospital, Taipei, Taiwan—*De-Kuang Hwang, Nai-Wen Fan; *The University of Sydney, Save Sight Institute, Sydney, Australia—*Hong Sheng Chiong, Javier Lacorzana, Maria Cabrera-Aguas, Stephanie Watson; *University of California Los Angeles Stein Eye Institute, Los Angeles, California, USA—*Edmund Tsui, Nina M. Cherian, Rachel Feit-Leichman, Reginald E. Hughes Jr, Tania Onclinx, Joseph K Privratsky; *University of California San Diego Shiley Eye Institute; La Jolla, California, USA—*Carol Yu, Esmeralda McClean, Iliana Molina; *University of California San Francisco Francis I. Proctor Foundation, San Francisco, California, USA—*Armin Hinterwirth, Cindi Chen, Danny Yu, Yu Heng Lu, Elodie Lebas, Emily Colby, Gerami Seitzman, Kevin Ruder, Lina Zhong, Michael Deiner, Thomas Abraham, Thomas Lietman, Thuy Doan (Principal Investigator), Travis Porco, Stephen McLeod; *University of California Berkeley School of Optometry, Berkeley, California, USA—*Kuniyoshi Kanai, Meredith Whiteside; *University of Nebraska Medical Center Truhlsen Eye Institute, Omaha, Nebraska, USA—*Steven Yeh, Tolulope Fashina; *University of New Mexico, Albuquerque, New Mexico—*James Chodosh; *University of Papua New Guinea School of Medicine and Health Sciences, Port Moresby, Papua New Guinea—*Bridgit Tarkap, Jambi N. Garap, Magdalene Mangot; *Vanuatu Eye Program, Ministry of Health, Vanuatu—*Edwin Amel, Fasihah Taleo, Johnson Kasso, Kalbule Willie, Madopule Nanu, Prudence Rymill; *World Health Organization—*Anthony W. Solomon.
